# Are Conventional Combined Training Interventions and Exergames Two Facets of the Same Coin to Improve Brain and Cognition in Healthy Older Adults? Data-Based Viewpoint

**DOI:** 10.2196/38192

**Published:** 2022-10-03

**Authors:** Jean-Jacques Temprado, Marta Maria Torre

**Affiliations:** 1 Aix Marseille Université Institut des Sciences du Mouvement Marseille France

**Keywords:** aging, older, gerontology, exergame, physical activity, cognition, training, intervention, cognitive, brain, older adult, motor skills, exercise, physical, motor, combined training

## Abstract

Combining physical, motor, and cognitive exercises is expected to be effective to attenuate age-related declines of brain and cognition in older adults. This can be achieved either by conventional interventions or by exergames. This paper aimed to determine whether conventional combined training and exergame interventions are two comparable ways for delivering combined training. In total, 24 studies on conventional training and 23 studies on exergames were selected and compared. A common framework was used to analyze both types of combined training interventions. Our analysis showed that conventional combined training interventions were more effective than separated physical and motor training to improve brain and cognition, while their superiority over cognitive training alone remains to be confirmed. Exergames scarcely led to cognitive benefits superior to those observed after physical, motor, or cognitive training alone. Thus, although both conventional training interventions and exergames allowed delivering combined training programs, they are not two facets of the same coin. Further studies that are more theoretically grounded are necessary to determine whether interventions delivered via exergames may lead to superior benefits compared to conventional separated and combined training interventions.

## Introduction

Delaying or attenuating age-related cognitive decline is critical for preserving autonomy and quality of life of the increasing number of older adults. It has been widely demonstrated that separate cognitive, aerobic, muscular, and motor training are effective in this respect [1]. Moreover, it has been suggested that their integration into combined training interventions (CTIs) might be more effective than separated training [2-4]. In this context and in view of the role played by cognitive stimulations in CTIs [5,6], exergames (ie, interactive video games that require participants to be physically active to play) might be even more effective than conventional combined training programs, since they conjugate the effects of physical and motor exercises [1,7] and those of video game training on cognitive performance [8,9]. However, until now, no study has systematically compared, within the same experimental protocols, the respective benefits of ‘conventional’ CTIs and exergames with regard to cognitive outcomes in healthy older adults. “A review of reviews” (ie, 3 reviews on conventional cognitive and motor training and 3 on exergames) [10] recently addressed this issue and reported conflicting results. Specifically, the benefits of conventional CTIs were found superior to those of separated training in 2 reviews, but the superiority of exergames over physical or cognitive training alone was unclear in the selected reviews.

This paper aims to go a step further by reporting the results of a detailed comparison of studies that used conventional CTIs and those that used exergames to improve brain and cognition in healthy older adults. To fulfill this objective, based on the framework developed in 2 recently published review papers dedicated to conventional CTIs [11] and exergames [12], we compiled the data of 47 studies to compare randomized controlled trials and controlled trials that have used either conventional combined training or exergames to improve cognitive functions (Table 1 and Table 2).

**Table 1:** Selected reviews and studies on conventional combined training interventions. Studies were classified as a function of the type of combined intervention.

Conventional combined training interventions				Studies
Reviews				Law et al [13], Wollesen and Voelcker-Rehage [14], Zhu et al [15], Lauenroth et al [3], Levin et al [16], Tait et al [17], Gheysen et al [2], Joubert and Chainay [18], Gavelin et al [19], Wollesen et al [20], Gallou et al [10], Gou et al [21]
**Studies**				
	**Sequential**			
		PCT^a^	Fabre et al [22], Legault et al [23] Shatil et al [24], Linde and Alfermann [25], Shah et al [26], Mc Daniel et al [27], Desjardins-Crépeau et al [28]	
		MCT^b^	Oswald et al [29]	
		MDT^c^	Pieramico et al [30], Van het Reve and de Bruin [31], Rahe et al [32], Rahe et al [33], Kalbe et al [34]	
	**Simultaneous**			
		PCT	Theill et al [35], Leon et al [36], Norouzi et al [37], Eggenberger et al [38], Eggenberger et al [39]	
		MCT	Hiyamizu et al [40], Marmeleira et al [41], Falbo et al [42]	
		MDT	Ansai et al [43], Yokoyama et al [44], Nishiguchi et al [45], Jardim et al [46]	

^a^PCT: physical-cognitive training.

^b^MCT: motor-cognitive training.

^c^MDT: multidomain training.

**Table 2:** Selected reviews and studies on exergames. Studies were classified as a function of the type of combined intervention.

Exergames interventions				Studies
Reviews				Zhang and Kaufman [47], Bleakley et al [48], Ogawa et al [49], Howes et al [50], Stanmore et al [51], Vázquez et al [52], Mansor et al [53], Stojan and Voelcker-Rehage [54], Gallou-Guyot et al [10], Wollesen et al [20], Gavelin et al [19], Sakaki et al [55], Soares et al [56]
**Studies**				
	**Sequential**			
		PCT^a^	—^b^	
		MCT^c^	Park and Yim [57]	
		MDT^d^	Kayama et al [58]	
	**Simultaneous**			
		PCT	Anderson-Hanley et al [59], Barcelos et al [60]	
		MCT	Schoene et al [61], Schoene et al [62], Gschwind et al [63], Schättin et al [64], Adcock et al [65], Carrasco et al [66], Eggenberger et al [39], Eggenberger et al [67], Huang [68], Li et al [69]	
		MDT	Maillot et al [70], Chuang et al [71], Ordnung et al [72], Guimaraes et al [73], Htut et al [74], Bacha et al [75], Peng et al [76], Moreira et al [77], Gouveia et al [78]	

^a^PCT: physical-cognitive training.

^b^Not available.

^c^MCT: motor-cognitive training.

^d^MDT: multidomain training.

## A Structured Framework for Analyzing Combined Training Interventions

**Figure 1:**
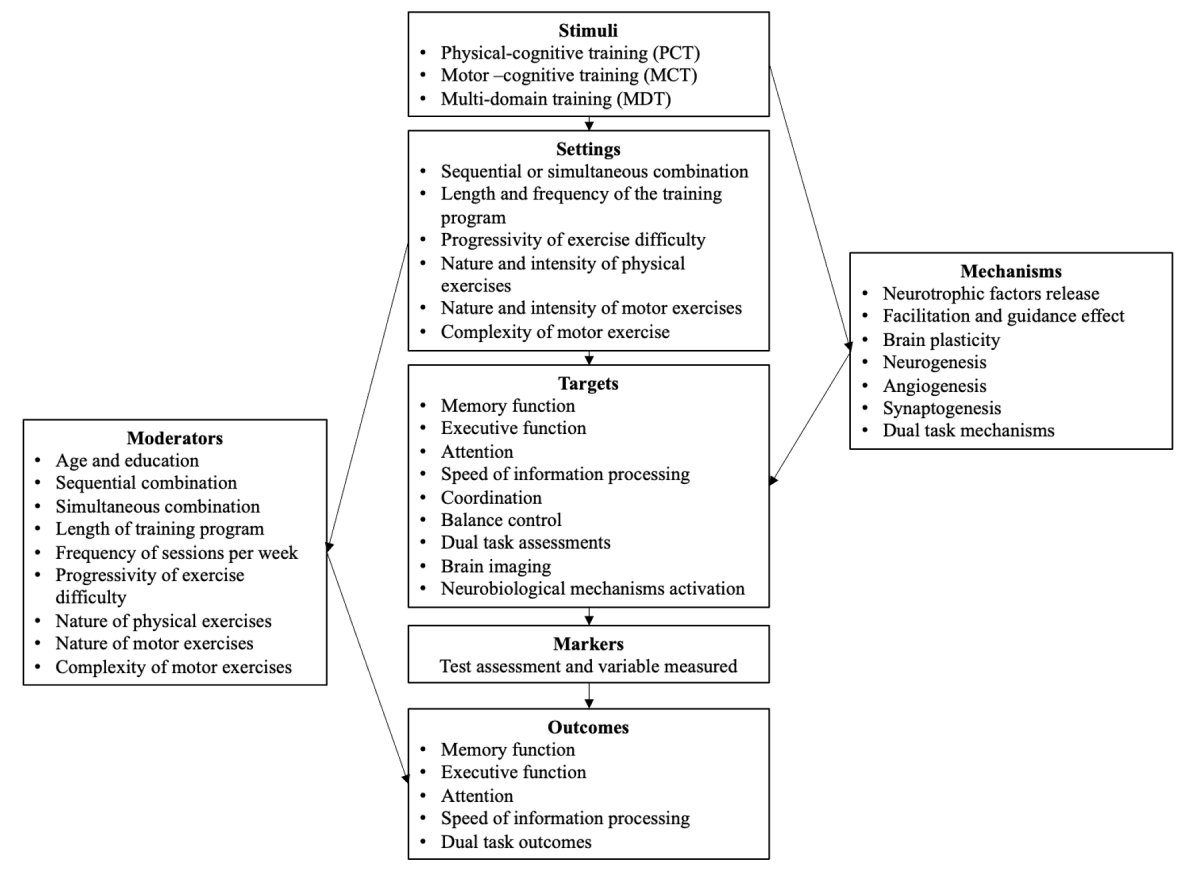
A multidimensional framework to analyze combined training interventions (detailed explanations are presented in a previous study [11]). Published under Creative Commons Attribution 4.0 International License.

We developed a framework to analyze CTIs, independent of whether they were delivered via conventional interventions or via exergames. Specifically, we distinguished the following: (1) the stimuli, which refer to different types of combined training; (2) the settings, which are the organizing features of training programs (ie, frequency, duration, intensity, instructions, feedback, individualization, and progressivity of increase in difficulty); (3) the targets of training, which were limited in this review to brain and cognitive levels, but other levels could be added in future works; (4) the markers, that is, the tasks and tests used to train or assess the participants, respectively; (5) the outcomes of different types of training, that is, the different variables that allow for quantifying the observed effects at brain and cognitive levels; (6) the moderators, who modulated the effects of training; and (7) the potential mechanisms, which were explicitly mentioned in different studies to predict and explain the effects of combined training (Figure 1).

Accordingly, 3 main training modes were distinguished: (1) physical-cognitive training (PCT), which correspond to the association of endurance (aerobic) and muscular resistance training and cognitive training, either sequentially or simultaneously; (2) motor-cognitive training (MCT), which refers to the association of complex motor skills training and cognitive training, implemented through the addition of cognitive tasks separated from the motor tasks (eg, mental calculation); and (3) multidomain training (MDT), which consists of associating aerobic exercises, complex motor skills, and cognitive tasks through laboratory-customized training situations. Notably, for conventional CTIs, we limited our analysis to randomized controlled trials and controlled trials in which it was possible to identify different training components (ie, physical, motor, and cognitive) that were associated with each other. Thus, according to this criterion, interventions implemented through natural motor activities (eg, tai chi, dance, or Nordic walking) were excluded. Although these activities included physical, motor, and cognitive components, their respective weights and levels of intensity or complexity were difficult to quantify. Based on this framework, in this study, we focused our comparative analysis on the following 4 main constructs: stimuli, settings, targets, and outcomes.

## The Database

Our analysis was grounded on the material included in 2 recently published reviews dedicated to conventional CTIs [11] and exergames [12], respectively. Specifically, 24 studies on conventional training and 23 studies on exergames, published from 2010 to November 2021, were selected on the basis of several criteria [11,12]. These studies were then analyzed to compare them according to the 4 chosen constructs of our framework (Table 1 and Table 2).

## Quantitative and Qualitative Differences Between Conventional Interventions and Exergames

### Stimuli

Motor and cognitive exercises were performed simultaneously in 100% (n=23) of the exergames studies, whereas sequential presentation of physical and cognitive exercises was used in 58% (7/12) of conventional PCT studies, 22% (2/9) of MDT studies, and 50% (2/4) of MCT studies. Thus, one can hypothesize that several studies on conventional CTIs used mechanisms that were different from those involved in exergame interventions. Another important issue concerns the distribution of the 3 training modes (ie, PCT, MCT, and MDT), which differed in conventional CTI and exergames studies. Indeed, the proportion of PCT studies was much higher for conventional interventions compared to exergaming (12/24, 50% and 2/23, 8%, respectively), whereas the inverse was observed for MCT 16% (4/24) for conventional interventions and 47% (11/23) for exergaming, respectively) and MDT studies 37% (9/24) for conventional studies and 43% (10/23) for exergaming, respectively). This resulted from the predominant use of commercial exergames (eg, Xbox Kinect and Wii Balance Board), which were cheaper than the stationary cybercycle used for implementing PCT in exergames studies [59,60]. It could be concluded from the distribution of PCT in both conventional and exergaming intervention studies that, on average, the latter was less physically demanding than the former; supporting evidence could be found in a study by Graves et al [79], and a discussion is presented in Gonçalves et al’s 2021 study [80]. However, this issue is a matter of debate, since few studies demonstrated that commercial exergames can be the support of intense physical activity [81,82], whereas others showed that they only facilitated light- to moderate-intensity physical activity [83]. On the other hand, since commercial exergames usually required upper limb movements, whole body movements, stepping, weight shifting or balance control, motor exercises supporting MCT and MDT in conventional CTIs and exergames studies were roughly similar.

### Settings

Conventional CTIs most frequently aimed to compare several groups simultaneously. Indeed, 21/24 (87%) involved a passive control group, alone or together with cognitive training (15/24, 62%) and physical or motor training (13/24, 54%) groups. In total, 8/24 (33%) of the conventional training studies involved 3 training groups (ie, CTI, physical or motor, and cognitive), whereas it was the case in only 1/23 (4.3%) of the exergames studies. Most frequently, exergames studies included 2 groups, that is, either a passive control group (9/23, 39.1%), a physical or motor training group (13/23, 56.5%), or less frequently, a cognitive training group (2/23, 8.6%), in addition to the exergaming group. In both conventional CTI and exergames studies, permanence and transfer of training effects were scarcely investigated, so no reliable conclusion can be drawn in this respect.

### Targets

No main difference was observed between the cognitive abilities tested in conventional CTIs and exergames studies. The most frequently tested were memory, executive functions, attention, and information processing speed, but there were no ‘a priori’ assumptions about the type of functions that could be affected more or less by each CTI. The respective effects of conventional combined training and exergames on brain health and neurobiological mechanisms cannot be reliably compared due to the small number of related studies and their heterogeneity (ie, 4 and 2 studies, respectively).

### Outcomes

#### Combined Training Versus Passive Control Groups

Independent of the training modes (ie, PCT, MCT, or MDT), positive effects were observed, relative to passive control groups, in all conventional CTIs and exergames studies, for at least one of the targeted cognitive functions, that is, memory, attention, executive functions, and information processing speed. These results were observed for both sequential and simultaneous associations between cognitive and physical or motor exercises. Unfortunately, it was impossible to determine whether cognitive functions were differently impacted by conventional combined training and exergames, respectively. These results are consistent with those reported by Gallou-Guyot et al [10]. Unsurprisingly, they suggest that regardless of how combined training is delivered (ie, conventional interventions or exergames, and PCT, MCT, or MDT), combined training programs always lead to superior benefits compared to inactivity.

#### Combined Training Versus Physical Training

In conventional CTIs, superior benefits of combined training over separated physical training were observed in 100% (n=13) of MCT and MDT studies and in most of the PCT studies (8/12, 66.6%). On the other hand, superior benefits of exergaming compared to conventional physical or motor training alone were observed in only one study on exergames [59], whereas no difference was found between the exergames and separated training groups in the 4 other studies (2 on MCT and 2 on MDT).

#### Combined Training Versus Cognitive Training

In conventional training studies, compared to cognitive training alone, superior benefits of CTIs were observed in almost one-third of PCT studies (n=4), but never in MCT and MDT studies. In the 2 studies that compared exergaming and cognitive training, one reported a superiority of the former over the latter on executive functions [67], whereas the other did not [74]. Thus, the number of related studies was too small to draw reliable conclusions about the superiority of exergames over cognitive training alone.

## Limitations and Study Comparisons

The studies including 4 training groups (ie, combined training, separated physical and cognitive training, and a control group), which could allow for a complete comparison, were scarce (ie, 8 conventional training studies out of 47, in total). A second observation was that despite the type of intervention (ie, conventional or exergames), the mechanisms underlying eventual differences with physical and cognitive training groups were rarely addressed in the reviewed studies. A third observation was that information relative to intensity and the nature of physical exercises, the nature and levels of complexity of motor exercises, and progressivity of difficulty was neglected in most studies, so it was impossible to estimate why physical or motor training was effective (or not) to improve physical, motor, or cognitive performance. This was the case, in particular, in exergames studies. In addition, in several studies, cognitive training procedures and (exer)game contents were not described or were only superficially described. Finally, due to the small number of studies available to support some comparisons (eg, exergames and cognitive training; conventional CTI vs exergames), the results remain to be confirmed or even established in future studies.

## Discussion

In this paper, we aimed to determine whether conventional CTIs and exergames were two comparable ways for delivering combined training. Our analysis showed that conventional CTIs were more effective than separated physical and motor training to improve brain and cognition, but their superiority over cognitive training alone remains to be confirmed in further studies. On the other hand, exergames scarcely led to cognitive benefits superior to those observed after physical, motor, or cognitive training alone. A plausible reason is that the existing exergames did not allow reaching high enough levels of physical effort [10] or motor skills complexity, and they used the resources of virtual reality and video games insufficiently to improve the cognitive load of different exercises [84]. This is not to say that exergame interventions cannot succeed in being more effective than conventional CTIs. However, further studies, grounded on theoretical knowledge provided by the literature on physical, motor, and cognitive training are necessary to determine whether interventions delivered via exergames may lead to superior benefits compared to separated and combined CTIs. In particular, since commercial exergames are not designed specifically for older adults, exergames studies should use new solutions that are more grounded on theoretical foundations [84].

Finally, this analysis showed that conventional CTIs and exergames studies did not address the same research questions, thereby precluding reliable comparisons of their benefits. Specifically, conventional CTI studies prominently aimed to compare benefits of separate training programs, whereas exergames studies focused on the benefits of exergaming per se, most often relative to inactive control groups. Thus, contrarily to our expectations, they seem to be separated domains of the literature on aging, which, until now, have developed independently of each other. In particular, the literature on exergames has not yet reached the level of maturity of those on conventional CTIs, which itself remains heterogeneous and suffers from methodological weakness and lack of a strong conceptual background [11,84,85]. Therefore, although they both allow for delivering combined training programs, conventional and exergames interventions are not two facets of the same coin; rather, they are two coins we do not know which is more valuable. Accordingly, future studies should aim to develop new exergames that would capitalize more on the knowledge from studies on conventional CTIs, particularly concerning the underlying mechanisms. These studies should also systematically compare the effectiveness of existing or new exergames and that of conventional CTIs.

## Abbreviations

CTI: combined training intervention
MCT: motor-cognitive training
MDT: multidomain training
PCT: physical-cognitive training
